# A competing risk nomogram predicting cause-specific mortality in patients with lung adenosquamous carcinoma

**DOI:** 10.1186/s12885-020-06927-w

**Published:** 2020-05-16

**Authors:** Xiao Wu, Wenfeng Yu, R. H. Petersen, Hongxu Sheng, Yiqing Wang, Wang Lv, Jian Hu

**Affiliations:** 1 Department of Thoracic Surgery, First Affiliated Hospital, College of Medicine, Zhejiang University, No. 79, Qingchun Road, Hangzhou, 310003 China; 2 Department of Cardiothoracic Surgery, Copenhagen University Hospital, Rigshospitalet, Copenhagen, Denmark

**Keywords:** Adenosquamous carcinoma, Lung cancer, Competing-risk analysis, Cumulative incidence, Nomogram

## Abstract

**Background:**

Adenosquamous carcinoma (ASC) is an uncommon histological subtype of lung cancer. The purpose of this study was to assess the cumulative incidences of lung cancer-specific mortality (LC-SM) and other cause-specific mortality (OCSM) in lung ASC patients, and construct a corresponding competing risk nomogram for LC-SM.

**Methods:**

Data on 2705 patients with first primary lung ASC histologically diagnosed between 2004 and 2015 were extracted from the Surveillance, Epidemiology, and End Results (SEER) database. The cumulative incidence function (CIF) was utilized to calculate the 3-year and 5-year probabilities of LC-SM and OCSM, and a competing risk model was built. Based on the model, we developed a competing risk nomogram to predict the 3-year and 5-year cumulative probabilities of LC-SM and the corresponding concordance indexes (*C*-indexes) and calibration curves were derived to assess the model performance. To evaluate the clinical usefulness of the nomogram, decision curve analysis (DCA) was conducted. Furthermore, patients were categorized into three groups according to the tertile values of the nomogram-based scores, and their survival differences were assessed using CIF curves.

**Results:**

The 3-year and 5-year cumulative mortalities were 49.6 and 55.8% for LC-SM and 8.2 and 11.8% for OCSM, respectively. In multivariate analysis, increasing age, male sex, no surgery, and advanced T, N and M stages were related to a significantly higher likelihood of LC-SM. The nomogram showed good calibration, and the 3-year and 5-year C-indexes for predicting the probabilities of LC-SM in the validation cohort were both 0.79, which were almost equal to those of the ten-fold cross validation. DCA demonstrated that using the nomogram gained more benefit when the threshold probabilities were set within the ranges of 0.24–0.89 and 0.25–0.91 for 3-year and 5-year LCSM, respectively. In both the training and validation cohorts, the high-risk group had the highest probabilities of LC-SM, followed by the medium-risk and low-risk groups (both *P* < 0.0001).

**Conclusions:**

The competing risk nomogram displayed excellent discrimination and calibration for predicting LC-SM. With the aid of this individualized predictive tool, clinicians can more expediently devise appropriate treatment protocols and follow-up schedules.

## Background

Adenosquamous carcinoma (ASC), a rare histological subtype of lung cancer, accounts for less than 3% of all lung cancer cases [1–4]. In general, ASC is defined as a carcinoma with an adenocarcinoma (ADC) component and a squamous cell carcinoma (SCC) component each exceeding 10% of the entire tumour [5]. There is a substantial difference between ASC and other histological subtypes of lung cancer regarding clinicopathological characteristics. ASC patients are more likely to present with a larger tumour size, a higher frequency of lymphatic/pleural invasion, and a worse histological grade than ADC and SCC patients [3, 6]. In terms of survival, ASC patients have an unfavourable prognosis compared to ADC and SCC patients [3, 6, 7].

The majority of ASC patients are diagnosed at an age over 60 years [3, 6, 7], and elderly patients tend to have a higher prevalence of comorbidities than younger patients [8]. In addition, the presence of comorbidities has been suggested to be a strong predictor of other cause-specific mortality (OCSM) in various cancers [9–11]. Considering the high risk of OCSM, it is essential to take OCSM into account when performing survival analysis for lung ASC. However, in the presence of competing events (such as OCSM), a traditional Cox proportional hazards model is no longer suitable, as it ignores the existence of competing risks, which may inevitably overestimate the incidence of cancer-specific mortality [12]. In this context, the competing risk model is superior to the conventional Cox model because it takes into consideration competing events and can differentiate between the effects of therapy and risk factors on specific events [12, 13]. However, to date, there are no studies that have adopted a competing risk model to examine the factors influencing the prognosis of patients with lung ASC.

In addition, nomograms, which provide a visual display of a linear prediction model, can be used to calculate the individual risk probabilities of a clinical event based on the predictive variables in the graph [14]. Because of their usefulness, nomograms have been extensively applied in various cancers, such as non-small-cell lung cancer (NSCLC) [15], hepatocellular carcinoma [16], and breast cancer [17]. However, to our knowledge, there are no studies using a competing risk regression model to develop a nomogram to predict the survival of lung ASC patients.

Therefore, a competing risk analysis was performed to determine the predictive factors for lung cancer-specific mortality (LC-SM) in patients with lung ASC. We developed a nomogram to offer clinicians a quantitative means to assess the individual cumulative incidences of LC-SM to improve clinical decision making.

## Methods

### Data sources

Data on patients with first primary lung ASC histologically diagnosed between 2004 and 2015 were extracted from the Surveillance, Epidemiology, and End Results (SEER) registries (1975–2016 dataset). The study population comprised patients with the International Classification of Diseases for Oncology, Third Edition (ICD-O-3) site code C340-C349 and histological code 8560/3. The study time span was set from 2004 to 2015 on the basis of the first year of the American Joint Committee on Cancer (AJCC) 6th edition (2004+) and a minimum of one-year follow-up.

**Fig. 1:**
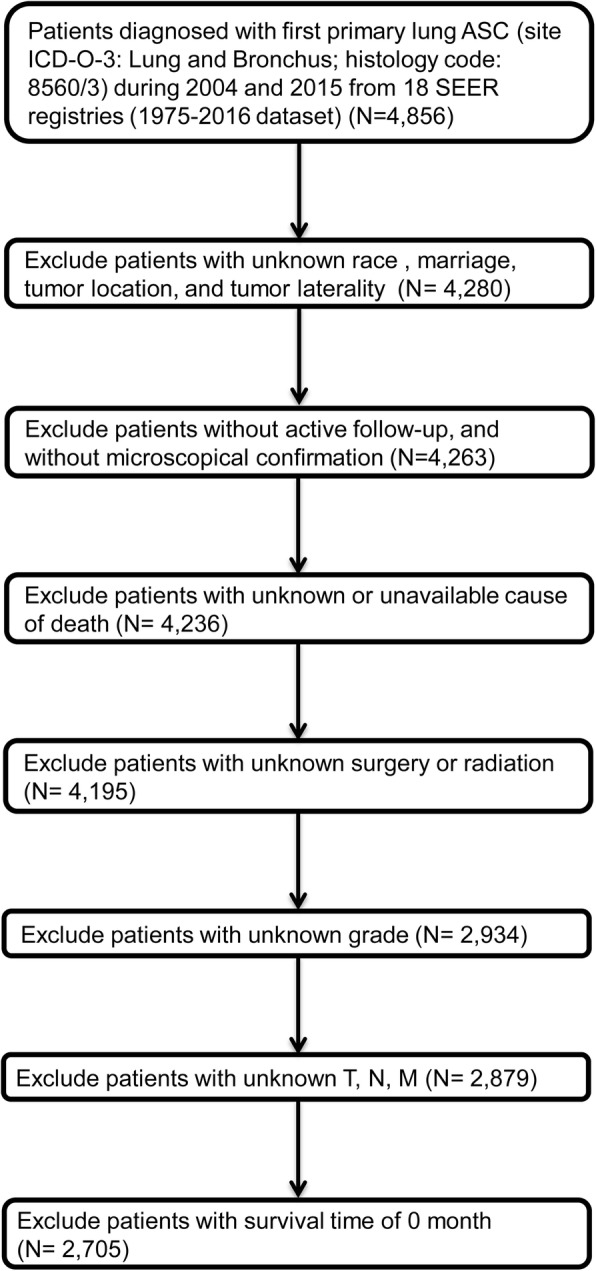
Flow diagram presenting the screening process in the SEER database

Related demographic and clinicopathological variables were collected, including age, sex, race, marital status, tumour laterality, tumour site, histological grade, TNM stage, surgical treatment, survival time, and causes of death. We excluded patients with any unknown variable values mentioned above. Age was categorized as < 65 years and ≥ 65 years; marital status was divided into unmarried and married; histological grade was classified into grade I (well differentiated), grade II (moderately differentiated), grade III (poorly differentiated), and grade IV (undifferentiated); and causes of death were categorized as alive, LC-SM and OCSM. As no radiation and unknown radiation have been merged into “None/Unknown” since 2016, and there was a substantial heterogeneity in chemotherapy, we did not include radiation and chemotherapy as variables in the present study. The detailed screening process is displayed in Fig. 1.

### Statistical analysis

Continuous variables with a normal distribution are expressed as the mean ± standard deviation (SD), and continuous variables with a skewed distribution are presented as the median (interquartile range, IQR). In the competing risk model, OCSM was regarded as a competing event for LC-SM. First, we computed the cumulative incidence function (CIF) for LC-SM and OCSM. Further subgroup analysis was carried out according to age, sex, race, marital status, tumour laterality, tumour site, histological grade, TNM stage, and surgery, and corresponding CIF curves were plotted for these variables. The significant differences in CIF values among subgroups were evaluated by Gray’s test [18]. Second, for the purpose of developing a competing risk regression model for LC-SM, the data set was randomly split into a training cohort (2/3) and a validation cohort (1/3). In total, 1803 cases serving as the training cohort were employed for model development, and 902 cases serving as the validation cohort were used for model validation. Third, variables that were perceived as clinically relevant beforehand or considered significant in the univariate analysis (*P* < 0.1) were introduced into a stepwise competing risk regression model. Subsequently, the optimal regression model was fitted when incorporating the predictive variables selected by the stepwise regression procedure. Fourth, we calculated the subdistribution hazard ratio (SHR) of the included variables for LC-SM based on the multivariate competing risk model, and a nomogram on the basis of the coefficients from the model was developed. To evaluate the model performance, the concordance index (C-index) was utilized to estimate the predictive accuracy (discrimination), and calibration curves (agreement between the observed probability and predicted probability at a certain time point) were constructed to assess the calibration with the aid of the R package “riskRegression” [19]. We also performed ten-fold cross validation for all data sets which were randomly partitioned into ten equal-sized subsamples [20]. Finally, decision curve analysis (DCA) was conducted to assess the clinical usefulness and net benefit of the competing risk model [21]. To determine whether the nomogram could successfully distinguish high-risk from low-risk lung ASC patients, each patient’s prediction score was derived according to the nomogram, and the patients were categorized into the high-risk, medium-risk, and low-risk groups based on the tertile values of the risk scores. Subsequently, the corresponding CIF curves of the three groups were plotted for the training set and validation set, and the significant differences in CIF were assessed using Gray’s test. All statistical analyses were carried out employing the R software version 3.5.2. A two-tailed *P < 0.05* was considered statistically significant.

## Results

The baseline characteristics of the whole study cohort are presented in Table 1. In general, a total of 2705 lung ASC patients were identified in the SEER database. For these patients, the median age at diagnosis was 69 years (IQR: 61–76). A larger proportion of patients were aged above 65 years (1776, 65.7%), male (1437, 53.1%), married (1590, 58.8%), and white (2248, 83.1%). The majority of tumours were located in the upper lobe (1651, 61.0%) and on the right side (1564, 57.8%). Most patients were diagnosed with histological grade III (64.7%), followed by grade II (31.7%), grade IV (2.3%), and grade I (1.3%). The distribution of AJCC stage was as follows: 42.2% had stage I, 12.3% had stage II, 23.4% had stage III, and 22.1% had stage IV. A total of 67.2% of patients received surgical treatment.

**Table 1:** Cumulative incidences of death among patients with ASC

Variables	Nt (%)	Ne (%)		LC-SM (%)			OCSM (%)	
			3-year (95% CI)	5-year (95% CI)	*P*	3-year (95% CI)	5-year(95% CI)	*P*
Total	2705	1897	49.6 (47.7–51.5)	55.8 (53.8–57.8)		8.2 (7.1–9.2)	11.8 (10.5–13.1)	
Age (years)								
median (IQR)	69 (61–76)	70 (62–77)						
< 65 years	929 (34.3)	608 (32.1)	49.2 (45.9–52.4)	55.0 (51.6–58.3)	0.275	5.1 (3.7–6.5)	8.0 (6.2–9.9)	< 0.001
≥ 65 years	1776 (65.7)	1289 (67.9)	49.9 (47.5–52.2)	56.3 (53.8–58.7)		9.8 (8.4–11.2)	13.9 (12.2–15.6)	
Sex					0.002			0.421
Female	1268 (46.9)	858 (45.2)	45.8 (43.0–48.6)	52.4 (49.5–55.3)		8.3 (6.8–9.9)	11.9 (10.0–13.8)	
Male	1437 (53.1)	1039 (54.8)	53.0 (50.4–55.6)	58.8 (56.1–61.5)		8.0 (6.6–9.5)	11.8 (10.0–13.6)	
Race					0.008			0.027
Black	262 (9.7)	195 (10.3)	56.8 (50.7–63.0)	62.9 (56.7–69.1)		7.0 (3.9–10.2)	12.4 (8.0–16.8)	
White	2248 (83.1)	1571 (82.8)	48.4 (46.3–50.5)	54.4 (52.2–56.6)		8.6 (7.4–9.8)	12.2 (10.8–13.7)	
Other race	195 (7.2)	131 (6.9)	54.3 (46.9–61.7)	63.1 (55.6–70.6)		4.3 (1.4–7.3)	6.0 (2.3–9.7)	
Marital status					0.018			0.455
Married	1590 (58.8)	1091 (57.5)	48.1 (45.5–50.6)	54.2 (51.6–56.8)		7.9 (6.5–9.2)	11.3 (9.7–13.0)	
Unmarried	1115 (41.2)	806 (42.5)	51.9 (48.9–54.9)	58.1 (55.1–61.2)		8.6 (6.9–10.3)	12.6 (10.5–14.7)	
Tumour site					< 0.001			0.213
Upper lobe	1651 (61.0)	1130 (59.6)	47.8 (45.3–50.3)	53.7 (51.2–56.3)		8 (6.7–9.4)	12 (10.3–13.7)	
Middle lobe	113 (4.2)	79 (4.2)	42.4 (33.1–51.7)	53.8 (43.7–63.8)		11.2 (5.1–17.2)	18.6 (10.6–26.6)	
Lower lobe	838 (31.0)	605 (31.9)	52.3 (48.8–55.7)	58.2 (54.7–61.7)		8.5 (6.5–10.4)	11.4 (9.1–13.7)	
Main bronchus	58 (2.1)	53 (2.8)	82 (71.6–92.5)	86.8 (76.9–96.7)		5.2 (0–11)	5.2 (0–11.0)	
Overlapping lesion	45 (1.7)	30 (1.6)	42.8 (28–57.6)	52.8 (37.5–68.0)		4.6 (0–11)	7.1 (0–15.0)	
Tumour laterality					0.131			0.165
Right	1564 (57.8)	1105 (58.2)	48.7 (46.2–51.2)	55.2 (52.6–57.8)		8.6 (7.2–10)	12.9 (11.1–14.7)	
Left	1135 (42.0)	787 (41.5)	50.8 (47.9–53.8)	56.4 (53.4–59.4)		7.6 (6–9.2)	10.4 (8.5–12.3)	
Bilateral	6 (0.2)	5 (0.3)	66.7 (22.2–111.2)	83.3 (43.8–100)		0 (0–0)	0 (0–0)	
Grade					< 0.001			< 0.001
Grade I	35 (1.3)	21 (1.1)	21.1 (6.8–35.3)	21.1 (6.8–35.3)		21.5 (7–36)	25.6 (9.6–41.5)	
Grade II	857 (31.7)	529 (27.9)	38.2 (34.8–41.5)	45.9 (42.4–49.5)		7.5 (5.7–9.3)	12.8 (10.4–15.3)	
Grade III	1751 (64.7)	1294 (68.2)	55.4 (53.1–57.8)	61.1 (58.7–63.5)		8.3 (6.9–9.6)	11.1 (9.5–12.6)	
Grade IV	62 (2.3)	53 (2.8)	60.1 (47.7–72.6)	62.1 (49.6–74.6)		8.2 (1.2–15.2)	12.2 (3.5–20.8)	
T stage					< 0.001			< 0.001
T1	736 (27.2)	402 (21.2)	26.8 (23.5–30.1)	32.8 (29.2–36.4)		9.4 (7.2–11.6)	16.3 (13.3–19.2)	
T2	1213 (44.8)	839 (44.2)	47.2 (44.4–50.1)	54.2 (51.3–57.2)		9 (7.4–10.7)	12.1 (10.2–14.0)	
T3	211 (7.8)	174 (9.2)	68.9 (62.5–75.3)	74.6 (68.4–80.9)		6.6 (3.1–10.1)	9.9 (5.5–14.4)	
T4	545 (20.1)	482 (25.4)	78.2 (74.6–81.7)	83.1 (79.7–86.5)		5.2 (3.3–7.1)	6.1 (4.0–8.2)	
N stage					< 0.001			< 0.001
N0	1475 (54.5)	892 (47.0)	33.8 (31.4–36.3)	40.6 (38.0–43.2)		9.6 (8–11.1)	14.9 (12.9–16.9)	
N1	362 (13.4)	245 (12.9)	51.5 (46.2–56.7)	58.6 (53.2–64.0)		5.6 (3.1–8)	8.3 (5.2–11.4)	
N2	711 (26.3)	617 (32.5)	73.8 (70.5–77.1)	79.2 (76.0–82.3)		7 (5.1–8.9)	8.3 (6.2–10.4)	
N3	157 (5.8)	143 (7.5)	85.1 (79.2–91.0)	87.3 (81.5–93.2)		6.6 (2.6–10.5)	6.6 (2.6–10.5)	
M stage					< 0.001			< 0.001
M0	2108 (77.9)	1344 (70.8)	39.8 (37.7–42)	46.9 (44.7–49.2)		8.9 (7.6–10.1)	13.3 (11.8–14.9)	
M1	597 (22.1)	553 (29.2)	84.2 (81.2–87.1)	87.1 (84.2–89.9)		5.8 (3.9–7.7)	6.5 (4.5–8.6)	
AJCC Stage					< 0.001			< 0.001
I	1142 (42.2)	624 (32.9)	23.9 (21.4–26.4)	31.1 (28.3–34.0)		11.2 (9.3–13.1)	17.2 (14.8–19.6)	
II	332 (12.3)	213 (11.2)	47.1 (41.6–52.6)	52.7 (47.1–58.4)		5.7 (3.1–8.3)	9.4 (6.0–12.8)	
III	634 (23.4)	507 (26.7)	64.9 (61.1–68.8)	72.8 (69.1–76.5)		6.3 (4.3–8.2)	8.4 (6.0–10.7)	
IV	597 (22.1)	553 (29.2)	84.2 (81.2–87.1)	87.1 (84.2–89.9)		5.8 (3.9–7.7)	6.5 (4.5–8.6)	
Surgery					< 0.001			< 0.001
No	886 (32.8)	806 (42.5)	82.1 (79.5–84.7)	85.9 (83.4–88.5)		7.9 (6.1–9.7)	8.8 (6.8–10.8)	
Yes	1819 (67.2)	1091 (57.5)	34.1 (31.8–36.3)	41.7 (39.3–44.0)		8.3 (7–9.6)	13.3 (11.6–15.0)	

Abbreviations: *N*_*t*_ total number, *N*_*e*_ number of death events, *ASC* adenosquamous carcinoma, *CI* confidence interval, *LC-SM* lung cancer-specific mortality, *OCSM* other cause-specific morality, *AJCC* American Joint Committee on Cancer, *IQR* interquartile range

**Fig. 2:**
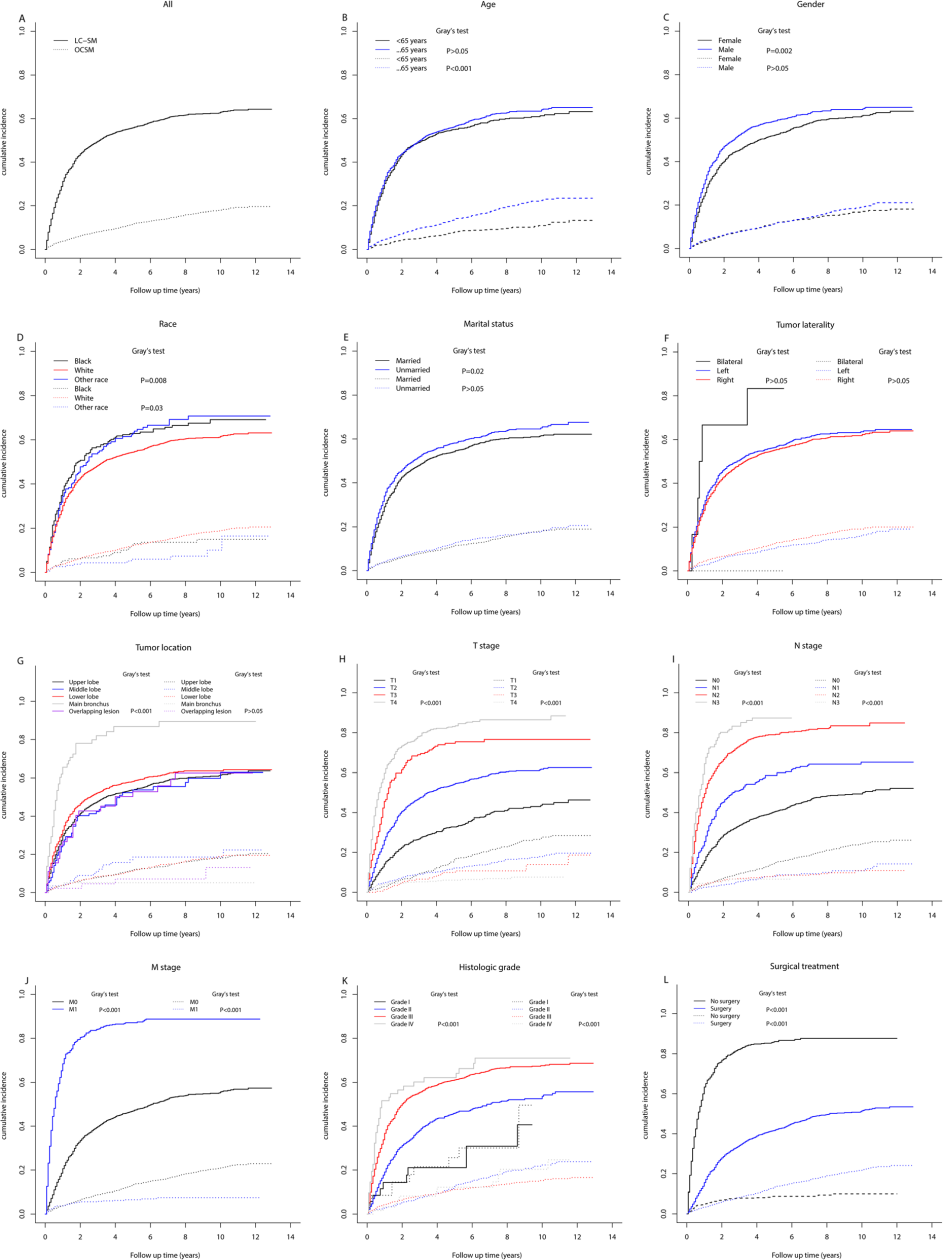
Cumulative incidence function curves of death for patients with lung ASC by different characteristics (solid line: LC-SM; dotted line: OCSM). Abbreviations: ASC: adenosquamous carcinoma; LC-SM: lung cancer-specific mortality; OCSM: other cause-specific morality

The median follow-up of the whole study cohort was 21 months (IQR: 8–52). In total, 1895 (70.1%) patients died throughout the whole follow-up period, of whom 1535 (81.0%) died due to lung cancer and 362 (19.0%) died due to non-lung cancer causes. The 3-year and 5-year cumulative incidences of LC-SM and OCSM by different clinicopathological characteristics are displayed in Table 1, and the corresponding CIF curves are presented in Fig. 2. Overall, the 3-year and 5-year LC-SM rates were 49.6% (CI: 47.7–51.5%) and 55.8% (CI: 53.8–57.8%), respectively, while the 3-year and 5-year OCSM rates were 8.2% (CI: 7.1–9.2%) and 11.8% (CI: 10.5–13.1%).

**Fig. 3:**
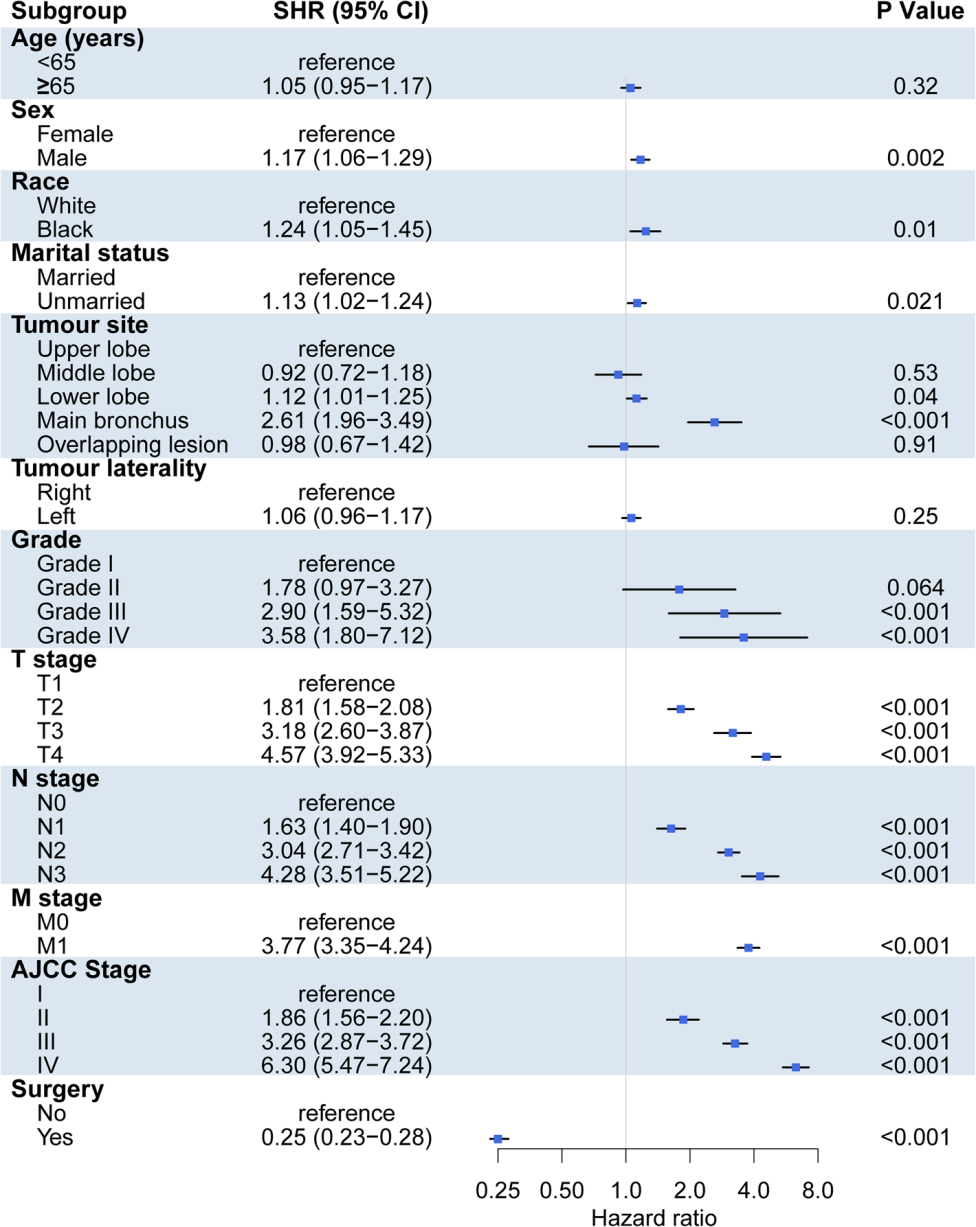
Forest plots visualizing the SHRs of the clinicopathological characteristics for LC-SM using the univariate competing risk model. Abbreviations: LC-SM: lung cancer-specific mortality; SHR: subdistribution hazard ratio

Subsequently, both univariate and multivariate competing risk models were adopted to evaluate the LC-SM of lung ASC patients. In univariate analysis, male sex, unmarried status, black race, main bronchus, advanced TNM stage, advanced histological grade, and surgical treatment were related to significantly higher incidences of LC-SM, whereas there were no significant differences for age and tumour laterality (Fig. 3**)**. In multivariate analysis, age, sex, surgery, T stage, N stage, and M stage were independent predictive factors for LC-SM (Table 2). In detail, increasing age was associated with an increased probability of LCSM. Male sex was related to a significantly higher likelihood of LCSM (1.26, CI: 1.10–1.43), while surgery was related to a significantly lower likelihood of LC-SM (0.45, CI: 0.37–0.53). Compared with patients with T1, advanced T-stage patients were more likely to face LCSM, with SHRs of 1.44 (1.21–1.72), 2.24 (1.72–2.92), and 1.99 (1.59–2.49) for T2, T3, and T4 patients, respectively. A similar phenomenon was observed among advanced N-stage patients compared with N0 patients, with SHRs of 1.52 (1.26–1.84), 1.57 (1.32–1.87), and 1.51 (1.12–2.03) for N1, N2, and N3 patients, respectively.

**Table 2:** Multivariate competing risk model for LC-SM in patients with lung ASC

Characteristics	Coefficient	SHR (95% CI)	P
Age	0.008		0.15
Age’	0.003		0.62
Sex			
Female	Reference	Reference	
Male	0.228	1.26 (1.13–1.40)	< 0.001
Surgery			
No			
Yes	−0.798	0.45 (0.39–0.52)	< 0.001
T stage			
T1	Reference	Reference	
T2	0.416	1.52 (1.30–1.77)	< 0.001
T3	0.817	2.26 (1.82–2.81)	< 0.001
T4	0.783	2.19 (1.82–2.63)	< 0.001
N stage			
N0	Reference	Reference	
N1	0.398	1.49 (1.26–1.76)	< 0.001
N2	0.5	1.65 (1.43–1.90)	< 0.001
N3	0.469	1.60 (1.28–2.00)	< 0.001
M stage			
M0	Reference	Reference	
M1	0.477	1.61 (1.40–1.86)	< 0.001

Abbreviations: *ASC* adenosquamous carcinoma, *CI* confidence interval, *LC-SM* lung cancer-specific mortality, *SHR* subdistribution hazard ratio

**Fig. 4:**
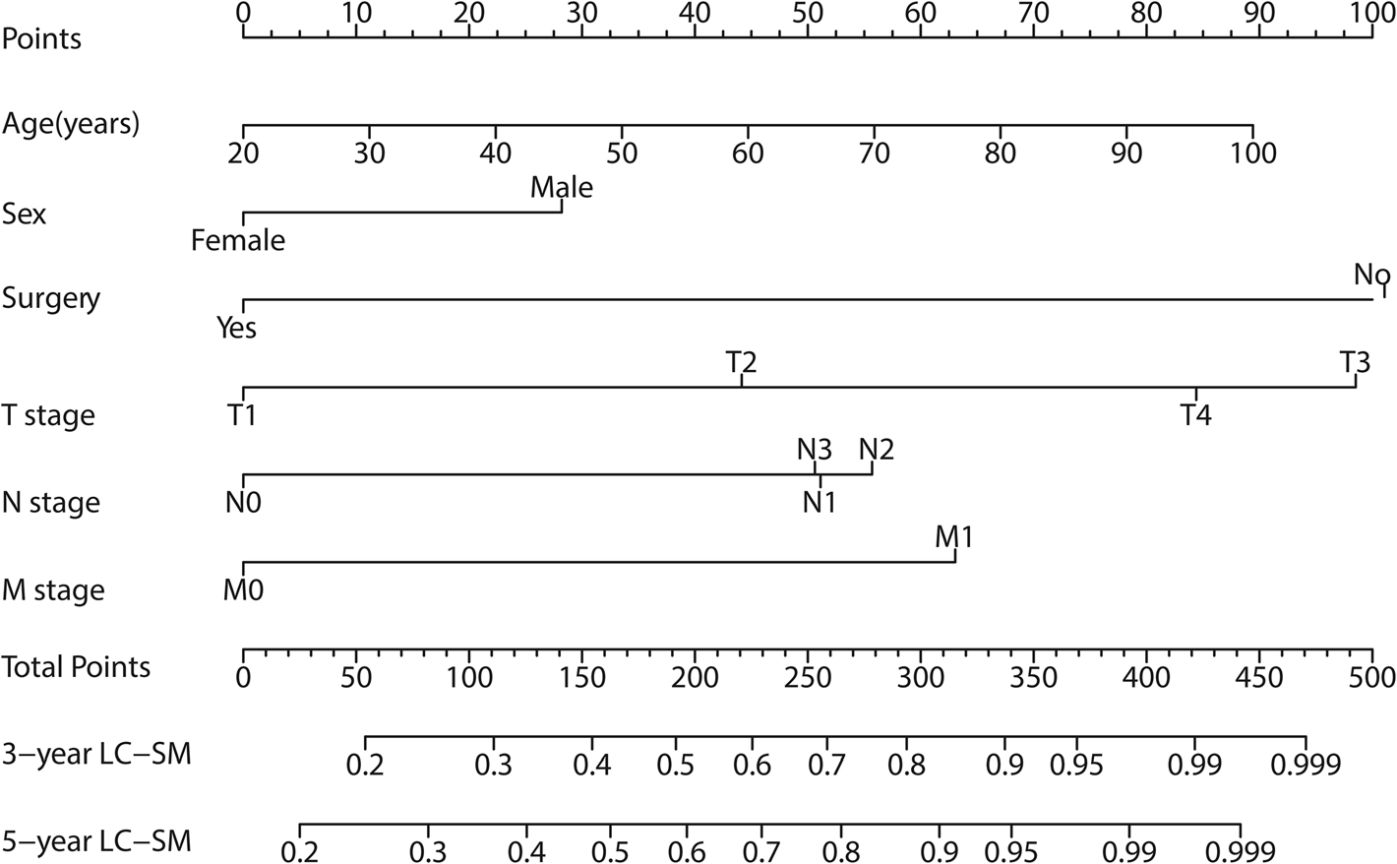
Competing risk nomogram predicting the 3-year and 5-year cumulative probabilities of death for LC-SM in patients with lung ASC. Abbreviations: ASC: adenosquamous carcinoma; LC-SM: lung cancer-specific mortality

A nomogram on the basis of the competing risk models was developed to calculate the 3-year and 5-year cumulative LC-SM probabilities (Fig. 4**)**. For each patient, first locate the values of different variables on the corresponding rows and then draw vertical lines pointing to the “Points” row to obtain corresponding scores. For instance, for a male patient, by drawing a vertical line straight up to the “Point” row, we would obtain approximately 28 points. Similarly, this process is performed for the other variables. By adding up these scores, a total score can be obtained and is located on the “Total Points” row. Subsequently, a vertical line can be drawn straight down to acquire the 3-year or 5-year cumulative death probabilities. For example, if the total score was 100, the corresponding 3-year and 5-year probabilities of LC-SM would be approximately 30 and 36%, respectively.

**Fig. 5:**
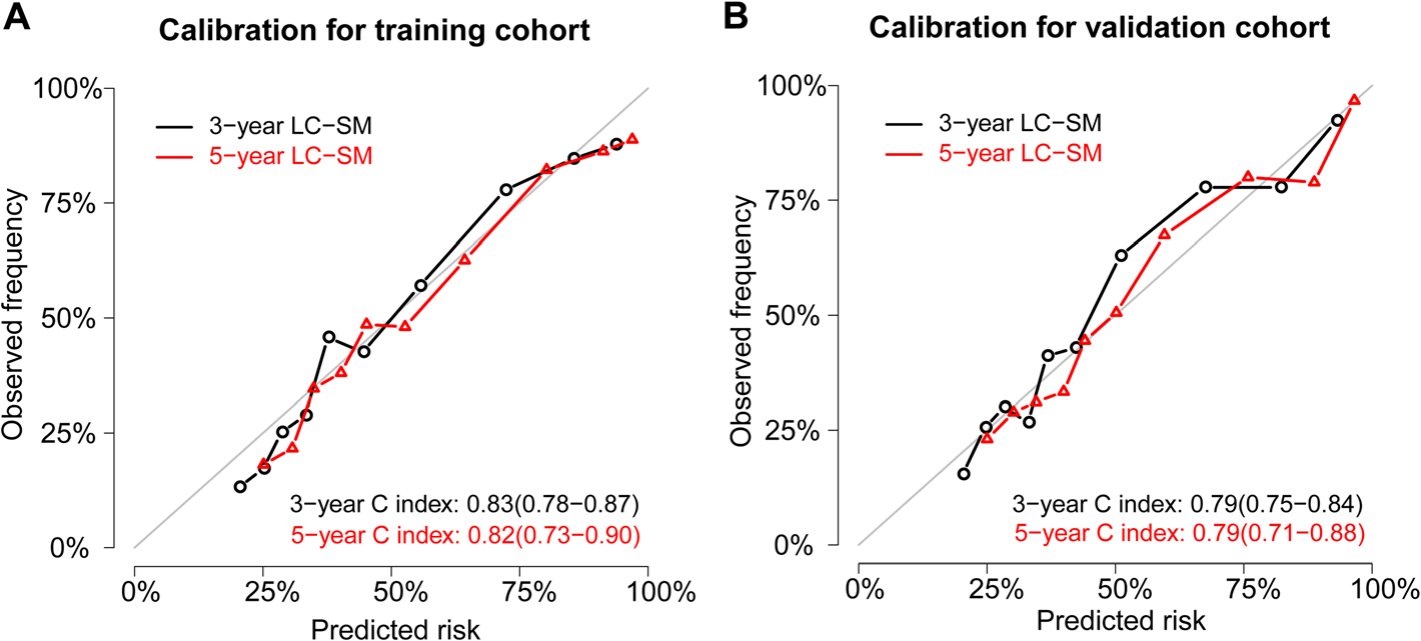
The 3-year and 5-year calibration curves accompanied by C-indexes for the training cohort and validation cohort. The X-axes represent the mean predicted mortality probability according to the prediction model. The Y-axes represent the observed cumulative incidence of mortality. The grey diagonal line indicates equality between the predicted and observed values. Abbreviations: LC-SM: lung cancer-specific mortality

The calibration curves accompanied by C-indexes are displayed in Fig. 5. As shown in Fig. 5, the calibration curves are close to the 45-degree diagonal line, indicating that the developed nomogram is well calibrated (good agreement between the observed mortality probability and the predicted mortality probability). Additionally, the 3-year and 5-year C-indexes for the nomogram predicting the probabilities of LC-SM were 0.83 (CI, 0.78–0.87) and 0.82 (CI, 0.73–0.90) for the training cohort, and 0.79 (CI, 0.75–0.84) and 0.79 (CI, 0.71–0.88) for the validation cohort, respectively, which indicated superb model discrimination. The ten-fold cross validation C-indexes are shown in Table 3. The adjusted 3-year and 5-year C-indexes were 0.81 (CI, 0.80–0.83) and 0.81 (CI, 0.80–0.83), respectively. Overall, the 3-year or 5-year C-indexes of the cross validation were almost equal to those of the training set or validation set, which indicated robust model performance.

**Table 3:** C-indexes of the predictive model for patients with ASC

Cohort	C-indexes		Adjusted C-indexes	
	3-year	5-year	3-year	5-year
Training cohort				
LC-SM	0.83 (CI, 0.78–0.87)	0.82 (CI, 0.73–0.90)		
Validation cohort				
LC-SM	0.79 (CI, 0.75–0.84)	0.79 (CI, 0.71–0.88)		
Overall cohort				
LC-SM			0.81 (CI, 0.80–0.83)	0.81 (CI, 0.80–0.83)

Note: Adjusted C-indexes of the model were calculated using ten-fold cross validation

Abbreviations: *ASC* adenosquamous carcinoma, *CI* confidence interval, *LC-SM* lung cancer-specific mortality

**Fig. 6:**
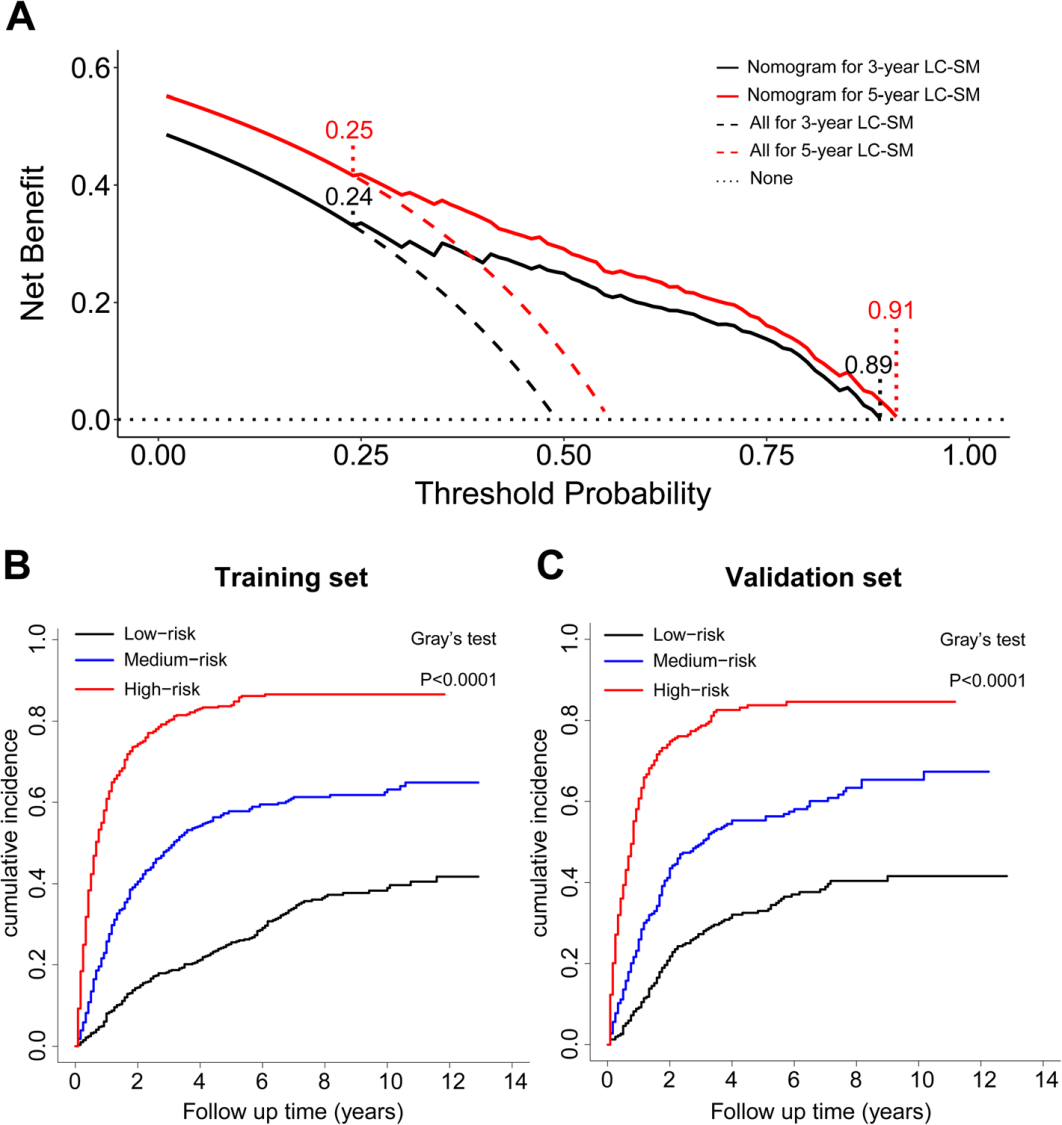
DCA based on the predictive model for the 3-year and 5-year LC-SM, and CIF curves of LC-SM among different risk groups. **a** The x-axis represents the threshold probability, and the y-axis represents the net benefit. The black and red dotted oblique lines reflect the assumption that all patients die due to LC-SM, and the black horizontal dotted line reflects the assumption that no patients die due to LC-SM. The black and red solid lines represent the threshold probability range, within which utilizing the nomogram to predict the LCSM gains more benefit than the hypothetical treat-all or treat-none scenarios. **b**-**c** CIF curves with the *P*-value of Gray’s test for the training cohort and validation cohort. Abbreviations: LC-SM: lung cancer-specific mortality; CIF: cumulative incidence function; DCA: decision curve analysis

The outcomes of DCA are shown in Fig. 6a, which shows that the clinical net benefit gained from the competing risk model was higher than that in the hypothetical non-screening or all-screening scenarios, when the threshold probabilities were within the range of 0.24–0.89 and 0.25–0.91 for 3-year and 5-year LCSM, respectively. According to the tertile values (117.1 and 180.5) of the nomogram-based scores derived from the training cohort, the patients were categorized into high-risk, medium-risk, and low-risk groups in the training cohort and validation cohort. As displayed in Fig. 6b-c, the high-risk group had the highest probabilities of LC-SM, followed by the medium-risk group and the low-risk group in the training cohort and validation cohort (both *P* < 0.0001). Therefore, when using the nomogram as a predictive tool, clinicians could successfully discriminate among different risk groups.

## Discussion

In the present study, a competing risk analysis was performed to investigate the predictive factors for LC-SM in patients with primary lung ASC from the SEER database. Of the 2705 total patients, 1535 (81.0%) died from lung cancer and 362 (19.0%) died from non-lung cancer causes. The 5-year CIFs for LC-SM and OCSM were 55.8 and 11.8%, respectively. We constructed a nomogram, which functions as a simple and useful clinical tool, to predict the individual probabilities of LC-SM for lung ASC patients, and the nomogram was demonstrated to have excellent clinical usefulness.

With regard to LC-SM, T stage, N stage, and M stage were significant independent predictive factors, unanimous with the eighth edition of the AJCC NSCLC staging system [22]. In addition, we also identified other important predictors, such as age, sex, and surgery, which have been incorporated into our nomogram. These predictors of an increased LC-SM, including advanced age, male sex, and surgery, have been proven in other studies [23, 24]. For example, a recent retrospective study investigating NSCLC based on the SEER database found that advanced age and male sex were related to decreased lung cancer-specific survival, and any form of surgical resection conferred a decreased risk of LC-SM [23]. H Zhou et al. analysed data from patients with radically resected stage I NSCLC in the SEER database and discovered that advanced age and male sex were correlated with a higher risk of cause-specific death [24]. As the AJCC staging system does not include important risk factors (including age, sex and surgery), the nomogram we developed was more discriminative and capable of providing a more accurate prognostic prediction for individual patients.

For the sake of patient counselling and clinical decision making, it is imperative to evaluate prognosis according to individual risk profiles. With the aid of a prognostic nomogram, clinicians can more expediently devise treatment protocols and follow-up strategies. Notably, competing risk nomograms have been developed for various cancers, such as nasopharyngeal carcinoma, breast cancer, gastrointestinal stromal tumours and melanoma [25–28]. However, as far as we know, this is the first study that constructed a competing risk nomogram based on a proportional subdistribution hazard model to predict the individual probabilities of LC-SM for lung ASC patients.

To assess the clinical usefulness of the nomogram, DCA was employed to determine whether the nomogram-based decisions could improve patients’ survival outcomes. Our findings showed that using the nomogram to predict LC-SM added more benefits than either the hypothetical treat-all-patients or treat-none scenarios as long as the threshold probabilities were within the range of 0.24–0.89 and 0.25–0.91 for 3-year and 5-year LCSM, respectively. In addition, with the assistance of the nomogram, clinicians could successfully discriminate among different risk groups, thereby making wiser clinical decisions. Therefore, the developed nomogram can be extremely useful in the processes of clinical practice.

The major strengths of the present study are that it had a large enough sample size and adopted a competing risk model to perform survival analysis. In general, the SEER database, covering approximately 27.8% of the US population, offers a sufficiently large sample to investigate predictive factors and further develop a model-based prognostic nomogram. Moreover, findings derived from the analysis based on the population-based database are more generalizable and representative than those from single-centre studies [29]. Moreover, the competing risk model fully takes into consideration any competing events, which renders the results more unbiased. Notably, the variables presented in the nomogram can be easily collected from routine medical records, so clinicians can more expediently predict cumulative death probabilities for lung ASC patients.

Undoubtedly, there are several limitations in this study. First, some known prognostic variables, such as cigarette smoking, chemotherapy, radiation therapy, and comorbidities, were not incorporated into the model. Thus, the nomogram only functions as a reference tool for clinicians to make clinical decisions. Further study is warranted to incorporate these variables into future research. Second, as the whole study population was from the US, the findings of the present study may not be generalizable to populations of other countries. Finally, although our model exhibits excellent performance in predicting the probabilities of LC-SM (with C-indexes fluctuating around 0.8), an external validation cohort including other patients is still necessary to demonstrate the model accuracy further.

## Conclusions

In conclusion, this is the first study using a competing risk model to evaluate the cumulative incidence of LC-SM for patients with lung ASC. We further developed a competing risk nomogram, and the nomogram displayed an excellent discrimination and calibration. With the aid of this individualized predictive tool, clinicians can more expediently devise appropriate treatment protocols and follow-up schedules.

## Data Availability

All the data of this study were derived from the SEER database, which was available from: www.seer.cancer.gov.

## Abbreviations

ASC: Adenosquamous carcinoma
ADC: Adenocarcinoma
SCC: Squamous cell carcinoma
OCSM: Other cause-specific mortality
NSCLC: Non-small-cell Lung Cancer
LC-SM: Lung cancer-specific mortality
SEER: Surveillance, Epidemiology, and End Results
ICD-O-3: International Classification of Diseases for Oncology, Third Edition
AJCC: American Joint Committee on Cancer
SD: Standard deviation
IQR: Interquartile range
CIF: Cumulative incidence function
C-index: Concordance index
DCA: Decision curve analysis
